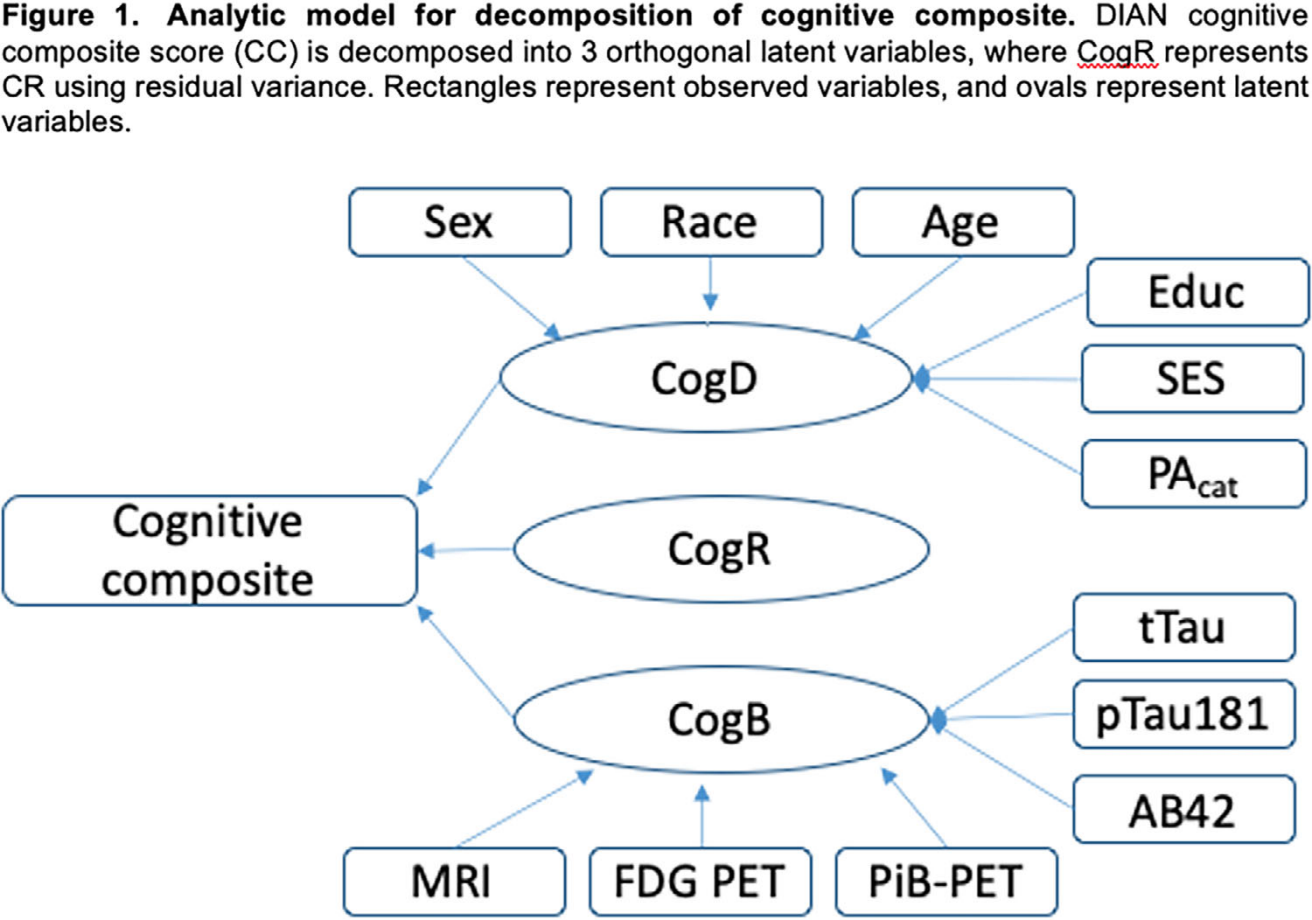# Cognitive reserve influences symptom onset and longitudinal decline in Dominantly Inherited Alzheimer's Disease

**DOI:** 10.1002/alz.086295

**Published:** 2025-01-03

**Authors:** Jorge J. Llibre‐Guerra, Ruijin Lu, Alan E. Renton, Brian A. Gordon, Tammie L.S. Benzinger, Andrew J. Aschenbrenner, Jason J. Hassenstab, Chengjie Xiong, Richard J. Perrin, Laura Ibanez, Alison M. Goate, Carlos Cruchaga, John C. Morris, Alisha Daniels, Eric McDade, Randall J. Bateman

**Affiliations:** ^1^ Washington University in St. Louis, School of Medicine, St. Louis, MO USA; ^2^ Ronald M. Loeb Center for Alzheimer’s Disease, Friedman Brain Institute, Icahn School of Medicine at Mount Sinai, New York, NY USA; ^3^ Washington University in St. Louis, St. Louis, MO USA; ^4^ Washington University School of Medicine in St. Louis, St. Louis, MO USA; ^5^ Washington University in St. Louis School of Medicine, St. Louis, MO USA

## Abstract

**Background:**

Cognitive reserve (CR) has emerged as a critical factor in understanding clinical‐cognitive heterogeneity in Alzheimer’s disease (AD). However, there is limited evidence of the effect of CR during asymptomatic phases of the disease, age at symptom onset (AAO) and longitudinal decline. In this study, we elucidate the impact of CR on AAO and decline rate using Dominantly Inherited Alzheimer’s Disease (DIAD) as a disease progression model.

**Methods:**

DIAD participants were selected from the Dominantly Inherited Alzheimer Network (DIAN) study^1^. Using structural equation models, we utilized a residual approach to quantify CR. Variance in DIAN cognitive composite^2^ was decomposed into three latent components (Figure 1): demographic component (CogD), biomarker component (CogB), and reserve component (CogR). CogD refers to variance in the cognitive composite explained by demographic factors. CogB refers to the variance explained by AD biomarkers and markers of neurodegeneration. CogR represents the residual variance in the cognitive composite not explained by CogD or CogB. We examined the relationship between these components and clinical outcomes, including AAO, Clinical Dementia Rating®‐Sum of Boxes (CDR®‐SB), and transition from asymptomatic (CDR = 0) to symptomatic (CDR>0).

**Results:**

319 participants (asymptomatic mutation carriers [aMC] n = 204, symptomatic MC [sMC] n = 115) were included in the analysis. Individuals with higher baseline CogR had greater odds ratio (OR) of remaining asymptomatic. For the aMC, after adjusting by mutation mean AAO^3^, one SD increase in the CogR component would multiply the OR of being CDR‐SB = 0 by 4.06 (95%CI 1.8‐8.9), while one SD increase in CogD and CogB component will multiply the OR by 2.6 (95%CI 1.1‐6.2) and 5.16 (95% CI 2.0‐13.3), respectively. For sMC, only the CogR and the CogB components were significant. One SD increase in CogR and CogB corresponds to 0.81 (95%CI 0.72‐0.92) and 0.60 (95%CI 0.50‐0.71) fold decrease in baseline CDR‐SB, respectively. Longitudinally, higher CogR values significantly predicted protection against conversion to CDR >0 and slower progression on CDR‐SB.

**Conclusions:**

CR significantly modifies both AAO and rate of clinical decline in DIAD. These findings highlight CR as a factor in AD progression and suggest potential avenues for intervention by enhancing CR to delay onset.